# *APP*, *PSEN1*, and *PSEN2* mutations in early-onset Alzheimer disease: A genetic screening study of familial and sporadic cases

**DOI:** 10.1371/journal.pmed.1002270

**Published:** 2017-03-28

**Authors:** Hélène-Marie Lanoiselée, Gaël Nicolas, David Wallon, Anne Rovelet-Lecrux, Morgane Lacour, Stéphane Rousseau, Anne-Claire Richard, Florence Pasquier, Adeline Rollin-Sillaire, Olivier Martinaud, Muriel Quillard-Muraine, Vincent de la Sayette, Claire Boutoleau-Bretonniere, Frédérique Etcharry-Bouyx, Valérie Chauviré, Marie Sarazin, Isabelle le Ber, Stéphane Epelbaum, Thérèse Jonveaux, Olivier Rouaud, Mathieu Ceccaldi, Olivier Félician, Olivier Godefroy, Maite Formaglio, Bernard Croisile, Sophie Auriacombe, Ludivine Chamard, Jean-Louis Vincent, Mathilde Sauvée, Cecilia Marelli-Tosi, Audrey Gabelle, Canan Ozsancak, Jérémie Pariente, Claire Paquet, Didier Hannequin, Dominique Campion, collaborators of the CNR-MAJ project

**Affiliations:** 1 Normandie Univ, UNIROUEN, Inserm U1245 and Rouen University Hospital, Department of Neurology and CNR-MAJ, Normandy Center for Genomic and Personalized Medicine, Rouen, France; 2 Department of Neurology, Orleans Regional Hospital, Orleans, France; 3 Normandie Univ, UNIROUEN, Inserm U1245 and Rouen University Hospital, Department of Genetics and CNR-MAJ, Normandy Center for Genomic and Personalized Medicine, Rouen, France; 4 Department of Neurology and CNR-MAJ, Lille University Hospital, Lille, France; 5 Inserm UMR-S 1171, Université Lille Nord de France, Lille, France; 6 Biochemistry Laboratory, Rouen University Hospital, Rouen, France; 7 Department of Neurology, Caen University Hospital, Caen, France; 8 Department of Neurology, Nantes University Hospital, Nantes, France; 9 Department of Neurology, Angers University Hospital, Angers, France; 10 Department of Neurology, Saint Anne University Hospital, Paris, France; 11 CNR-MAJ, AP-HP, Hôpital de la Pitié-Salpêtrière, Paris, France; and ICM, Inserm U1127, CNRS UMR 7225, Sorbonne Universités, UPMC-P6 UMR S 1127 - Hôpital Pitié-Salpêtrière, Paris, France; 12 Department of Neurology, Nancy University Hospital, Nancy, France; 13 Department of Neurology, Dijon University Hospital, Dijon, France; 14 Aix Marseille University, Inserm, INS, Institut de Neurosciences des Systèmes, Marseille, France; AP-HM, Service de Neurologie et Neuropsychologie, CHU Timone, Marseille, France; 15 Department of Neurology, Amiens University Hospital Center, Amiens, France; 16 Department of Neurology and CMRR Lyon University Hospital, Lyon, France; 17 Department of Neurology, Bordeaux University Hospital, Bordeaux, France; 18 Department of Neurology, Besançon University Hospital, Besançon, France; 19 Biochemistry Laboratory, Lille University Hospital, Lille, France; 20 Department of Neurology, Grenoble University Hospital, Grenoble, France; 21 Department of Neurology, Montpellier University Hospital, Montpellier, France; 22 Department of Neurology, Toulouse University Hospital, Toulouse, France; 23 CMRR Paris Nord AP-HP, Hôpital Lariboisière, INSERM, U942, Université Paris Diderot, Sorbonne Paris Cité, UMRS 942, Paris, France; 24 Department of Research, Centre Hospitalier du Rouvray, Sotteville-lès-Rouen, France; University of California San Francisco Memory and Aging Center, UNITED STATES

## Abstract

**Background:**

*Amyloid protein precursor* (*APP*), *presenilin-1* (*PSEN1*), and *presenilin-2* (*PSEN2*) mutations cause autosomal dominant forms of early-onset Alzheimer disease (AD-EOAD). Although these genes were identified in the 1990s, variant classification remains a challenge, highlighting the need to colligate mutations from large series.

**Methods and findings:**

We report here a novel update (2012–2016) of the genetic screening of the large AD-EOAD series ascertained across 28 French hospitals from 1993 onwards, bringing the total number of families with identified mutations to *n* = 170. Families were included when at least two first-degree relatives suffered from early-onset Alzheimer disease (EOAD) with an age of onset (AOO) ≤65 y in two generations. Furthermore, we also screened 129 sporadic cases of Alzheimer disease with an AOO below age 51 (44% males, mean AOO = 45 ± 2 y). *APP*, *PSEN1*, or *PSEN2* mutations were identified in 53 novel AD-EOAD families. Of the 129 sporadic cases screened, 17 carried a *PSEN1* mutation and 1 carried an *APP* duplication (13%). Parental DNA was available for 10 sporadic mutation carriers, allowing us to show that the mutation had occurred de novo in each case. Thirteen mutations (12 in *PSEN1* and 1 in *PSEN2*) identified either in familial or in sporadic cases were previously unreported. Of the 53 mutation carriers with available cerebrospinal fluid (CSF) biomarkers, 46 (87%) had all three CSF biomarkers—total tau protein (Tau), phospho-tau protein (P-Tau), and amyloid β (Aβ)_42_—in abnormal ranges. No mutation carrier had the three biomarkers in normal ranges. One limitation of this study is the absence of functional assessment of the possibly and probably pathogenic variants, which should help their classification.

**Conclusions:**

Our findings suggest that a nonnegligible fraction of *PSEN1* mutations occurs de novo, which is of high importance for genetic counseling, as *PSEN1* mutational screening is currently performed in familial cases only. Among the 90 distinct mutations found in the whole sample of families and isolated cases, definite pathogenicity is currently established for only 77%, emphasizing the need to pursue the effort to classify variants.

## Abstract 2

In a cohort including patients with familial Alzheimer disease as well as sporadic cases of early-onset Alzheimer disease, Dominique Campion and colleagues identify previously unreported mutations to *PSEN1* and *PSEN2*.

## Author summary

**Why was this study done?:**

- Mutations in the *amyloid protein precursor* (*APP*), *presenilin-1* (*PSEN1*), and *presenilin-2* (*PSEN2*) genes are a known cause of familial, early-onset Alzheimer disease (EOAD) (onset below age 65).
- However, in order to improve genetic counseling, it is necessary to report mutational screening from large cohorts of patients.

**What did the researchers do and find?:**

- In the present study, we performed sequencing of the *APP*, *PSEN1*, and *PSEN2* genes in EOAD families and in 129 sporadic cases.
- Mutations were identified in 170 EOAD families and in 18 sporadic cases.
- In 10 sporadic cases, we showed that the mutation was absent in the parents, indicating that it occurred “de novo.”

**What do these findings mean?:**

- Sufficient evidence of pathogenicity is reached for 77% of the 90 distinct mutations identified in this sample, allowing their use in genetic counseling.
- Our results suggest a potential benefit to screening nonfamilial Alzheimer disease (AD) cases with onset before 50 y for *APP*, *PSEN1*, and *PSEN2* mutations.

## Introduction

Alzheimer disease (AD) (MIM #104300) is the most common form of dementia. However, early-onset AD (EOAD) constitutes a minority of patients, with an estimated prevalence of 41.2 per 100,000 persons at risk [1]. Among these forms, *presenilin-1* (*PSEN1*) (MIM #104311), *presenilin-2* (*PSEN2*) (MIM #600759) [2–5], and *amyloid protein precursor* (*APP*) (MIM #104760) mutations [6–8] and duplications [9] cause autosomal-dominant EOAD (AD-EOAD), the prevalence of which is estimated to be 5.3 per 100,000 persons at risk [1]. *PSEN1* is the most commonly involved gene, with 221 mutations reported as pathogenic in the Alzforum database (www.alzforum.org/mutations). The second most commonly involved gene is *APP*, with 32 pathogenic mutations described, while 19 different *PSEN2* pathogenic mutations have been reported. *APP* encodes the amyloid-β precursor protein, the processing of which by the β-secretase and the γ-secretase complex leads to the production of the amyloid β (Aβ) peptide, a key event in AD pathogeny. The aggregation of the Aβ peptide in the brain’s parenchyma indeed triggers a cascade of events leading to AD. Its aggregation in cerebromeningeal vessels leads to cerebral amyloid angiopathy (CAA), a condition frequently associated with AD and responsible for recurrent haemorrhagic strokes and white matter lesions. *PSEN1* and *PSEN2* encode the presenilins, which constitute the catalytic subunit of the γ-secretase complex (for review, see [10,11]). AD-EOAD causative mutations are thought to be responsible for the increased aggregation of the Aβ peptide in the brain’s parenchyma through one of the two following mechanisms: increased overall production of all Aβ species (e.g., *APP* duplications or *APP* mutations located around the β cleavage site) or production of a more aggregation-prone form of the Aβ peptide.

The power to detect genetic variations has dramatically improved over the last few years, but the interpretation of rare variants remains a challenge in a high proportion of cases. The pathogenicity of most *APP*, *PSEN1*, and *PSEN2* variants has not yet been assessed through in vitro functional experiments. In cases of insufficient genetic evidence (i.e., lack or limited familial segregation or recurrence), definite pathogenicity of a given variant may therefore remain uncertain. An algorithm was proposed to classify those variants, based on (i) intrafamilial segregation, (ii) recurrence of the mutation in independent cases and association in case–control samples, (iii) residue conservation between *PSEN1* and *PSEN2* and residue localization on functional domains, and (iv) functional tests, when available [12]. Reporting patients carrying novel as well as previously known mutations along with the associated phenotypes will aid in classification of these variants and will eventually allow genetic counseling and inclusion in preventive trials for presymptomatic carriers [13].

We had previously described the *PSEN*s and *APP* mutational spectrum in a large French series of families with an EOAD diagnosis in at least two first-degree relatives from two generations [14]. The aim of the present article is to report mutations in additional families included since our last 2012 update [14]. Furthermore, we add the results of the genetic screening of 129 sporadic EOAD patients with an age of onset (AOO) before 51. The involvement of *PSEN1*, *PSEN2*, and *APP* mutations in the genetics of sporadic EOAD has been scarcely studied. In particular, systematic genetic assessments of series of patients with youngest AOO who are at high risk to carry an AD-EOAD mutation were not reported before. In these patients, the family history can remain negative because of a censoring effect (i.e., death of the transmitting parent before EOAD onset) [15] or if the mutation occurs de novo (i.e., if it is not found in parents but occurs in the parental germline or as a postzygotic event) [16].

## Materials and methods

The study was approved by the Paris Ile de France II ethics committee.

### Subjects

EOAD subjects were referred to the National Reference Center for Early-Onset Alzheimer Patients (CNR-MAJ) from 28 university hospitals across France. For each patient, AD diagnosis was established using the National Institute of Aging–Alzheimer’s Association (NIA–AA) criteria [17]. All patients underwent a comprehensive clinical examination, including personal medical and family history and neuropsychological assessment. Search for mutations in *APP*, *PSEN1*, and *PSEN2* genes was performed (i) in AD-EOAD presentations (i.e., if at least two first-degree relatives suffered from EOAD [AOO ≤65 y]) in two generations or (ii) in sporadic presentations if a patient without family history of AD had an age of onset before 51 y. No other exclusion criteria were applied. Familial cases (*n* = 63 mutation carriers belonging to 53 families, 42% males, mean AOO = 48 ± 5 y) were included in the 2012–2016 interval, whereas sporadic cases (*n* = 129, 44% males, mean AOO = 45 ± 2 y) were included from 1999 onwards. All patients were from European origin with the exception of five patients from African descent: three familial and two sporadic cases. Cerebrospinal fluid (CSF) AD biomarkers were assessed in 65% of the mutation carriers, and neuropathological examination was performed in 3 mutation carriers. A written consent to participate to the study was signed by every patient.

### CSF analysis

CSF samples were obtained using a Sprotte needle in polypropylene collection tubes and aliquoted after centrifugation into polypropylene tubes (catalog number 62.610.201; Sarstedt, Nümbrecht, Germany), then frozen at −80°C within 1 h. Aβ_42_, Tau, and P-Tau measurements were performed using enzyme-linked immunosorbent assays (ELISA) (Fujirebio Europe N.V., Ghent, Belgium) according to the manufacturer’s instructions. The analysis of all biomarkers was performed in two duplicates and averaged for statistical analyses. Following values were used to define biochemical AD signature: Aβ_42_ < 700 pg/mL; Tau > 350 pg/mL, and P-Tau > 60 pg/mL. Each subject was classified according to the Paris, Lyon, Marseille (PLM) scale [18]: class 0, corresponding to no pathologic biomarkers; class 1, corresponding to 1/3 pathologic biomarkers; class 2, corresponding to 2/3 pathologic biomarkers; and class 3, with all three biomarkers being pathologic.

### Genetic analyses

Genetic analyses were performed on DNA extracted from whole blood. Exons 2–12 of *PSEN1* (NM_000021.3), exons 4–13 of *PSEN2* (NM_000447.2), and exons 16 and 17 of *APP* (NM_000484.3) were analysed by Sanger sequencing. *APP* duplications and *PSEN1* exon 9/10 deletion were detected using QMPSF (quantitative multiplex PCR of short fluorescent fragments). *APOE* genotype was determined for each subject by Sanger sequencing. Primers are available upon request. Guerreiro’s algorithm [12] and Alzforum (www.alzforum.org/mutations) database were used to classify each mutation’s pathogenicity.

In sporadic cases, when DNA was available for both unaffected parents, parenthood was checked using a package of four microsatellites markers, each with a heterozygosity index from 79 to 88%, and the presence of the mutation identified in the proband was assessed by Sanger sequencing.

## Results

### Update of the EOAD French series

We identified mutations in 53 previously unreported AD-EOAD families and in 18/129 sporadic cases, including 44 *PSEN1*, 2 *PSEN2*, and 20 *APP* mutations as well as five *APP* duplications. The total number of mutation carriers including affected relatives in AD-EOAD families was *n* = 81 patients (Tables 1–4). Overall, 12 *PSEN1* mutations and 1 *PSEN2* mutation were previously unreported (Tables 1 and 2, in bold). In the next sections, we describe the mutation spectrum, with a particular focus on novel mutations.

**Table 1:** Previously unreported French families with AD-EOAD and sporadic cases carrying a *PSEN1* mutation. Novel mutations appear in bold.

Protein change	Nucleotide change	Exon	Pathogenicity	ID fam	APOE	AOO (years)	DD (years)	Family history	MC (*n*)	De novo
p.Ala79Val	c.236C>T	4	definite	EXT 85	E3 E4	[60–80]	[7–10]	F	1	
				ROU 252	E3 E4	[63–65]	[3–12]	F	3	
**p.Met84Thr**	**c.251T>C**	**4**	**definite**	**EXT 1117**	**E2 E4**	**[52–60]**	**[2–7]**	**F**	**2**	
**p.Pro88His**	**c.263C>A**	**4**	**probable**	**EXT 890**	**E3 E3**	**[42–45]**	**[2–5]**	**F**	**1**	
**p.Gly111Trp**	**c.331G>T**	**4**	**probable**	**EXT 502**	**E3 E3**	**47**	**4**	**S**	**1**	**Y**
p.Thyr115Cys	c.344A>G	5	definite	EXT 755	E3 E3	[44–50]	[3–9]	F	1	
**p.Pro117Gln**	**c.350C>A**	**5**	**probable**	**EXT 851**	**E2 E3**	**37**	**2**	**S**	**1**	**Y**
p.Met139Lys*	c.416T>A	5	probable	ALZ 034	E3 E3	37	10	S	1	Y
p.Ile143Thr	c.428T>C	5	definite	EXT 670	E3E4	35	6	S	1	U
p.Met146Ile	c.438G>A	5	definite	EXT 622	E3 E4	[42–43]	[1–7]	F	2	
p.His163Arg	c.488A>G	6	definite	EXT 766	E3 E4	[40–46]	[3–10]	F	1	
				EXT 1242	E3 E3	34	4	S	1	Y
p.Leu173Trp	c.518T>G	6	probable	EXT 149	E3E3	34	4	S	1	Y
**p.Ile180Asn**	**c.539T>A**	**6**	**possible**	**CAE 007**	**E4 E4**	**50**	**6**	**S**	**1**	U
**p.Phe205_Gly206 delinsCys**	**c.614_616del**	**7**	**probable**	**EXT 177**	**E2 E3**	**42**	**5**	**S**	**1**	**Y**
p.Gly206Asp	c.617G>A	7	definite	EXT 507	E3 E3	[30–32]	[2–8]	F	1	
**p.Met210Arg**	**c.629T>G**	**7**	**probable**	**EXT 832**	**E3 E3**	**[47–48]**	**[3–4]**	**F**	**1**	
p.Gly217Asp	c.650G>A	7	definite	ROU 1551	E3 E3	50	5	S	1	U
p.Gln222His	c.666G>C	7	definite	EXT 807	E2 E3	46	4	S	1	U
p.Ala231Thr	c.691G>A	7	definite	EXT 680	E2 E4	50	3	S	1	U
p.Met233Thr	c.698T>C	7	definite	EXT 1201	E3 E4	[44–45]	[2–4]	F	1	
p.Met233Ile*	c.699G>C	7	definite	MON 001	E3 E3	28	8	S	1	Y
**p.Phe237Cys**	**c.710T>G**	**7**	**probable**	**ROU 128**	**E3 E3**	**25**	**14**	**S**	**1**	**U**
p.Phe237Leu	c.711T>A	7	definite	EXT 1127	E3 E3	[47–48]	[2–4]	F	1	
**p.Leu241Arg**	**c.722T>G**	**7**	**probable**	**EXT 504**	**E3 E3**	**44**	**2**	**S**	**1**	**U**
p.Ala246Pro	c.736G>C	7	definite	EXT 1194	E2 E4	[50–51]	[1–4]	F	1	
p.Cys263Phe	c.788G>T	8	definite	EXT 1193	E3 E4	[48–53]	[7–8]	F	1	
				EXT 768	E3 E4	[55–65]	[1–3]	F	1	
p.Pro264Leu	c.791C>T	8	definite	EXT 966	E3 E3	50	6	F	1	
				EXT 1010	E3 E3	[55–65]	[4–5]	F	1	
				EXT 384	E3 E3	[52–60]	[4–8]	F	1	
				EXT 408	E3 E4	[41–55]	[13–16]	F	1	
				EXT 392	E3 E3	[50–58]	[4–11]	F	1	
p.Arg269His	c.806G>A	8	definite	EXT 1228	E3E3	60	[4–15]	F	1	
p.Glu273Gly	c.818A>G	8	definite	EXT 886	E3 E4	[46–53]	[4–7]	F	1	
				EXT 1195	E3 E4	[44–54]	[3–5]	F	1	
**p.Ala360Thr**	**c.1078G>A**	**10**	**possible**	**SAL 629**	**E3 E3**	**45**	**4**	**S**	**1**	**U**
p.Gly378Glu	c.1133G>A	11	probable	EXT 390	E3 E3	[38–44]	[6–9]	F	1	
p.Gly378Val	c.1133G>T	11	definite	EXT 596	E3 E3	[48–53]	[2–4]	F	2	
**p.Leu383Trp**	**c.1148T>G**	**11**	**probable**	**EXT 1071**	**E3 E3**	**[47–57]**	**[4–7]**	**F**	**1**	
p.Val391Phe	c.1171G>T	11	definite	EXT 902	E3 E3	[40–47]	[1–5]	F	2	
p.Leu418Phe	c.1254G>C	12	definite	ROU 1306	E3 E3	33	8	S	1	Y
p.Ser290_Ser319delinsCys (Δ 9)*	c.869-2A>G	9	definite	EXT 235	E2 E3	46	6	S	1	Y
**p.Ser290_Arg377delinsTrp (Δ 9–10)**	**c.(868+1_8691)_ (1129+1_1130–1)del**	**9 10**	**probable**	**EXT 313**	**E2 E4**	**[55–56]**	[5–6]	**F**	**1**	
**Total and ranges:**				**44**		**[25–80]**	**[1–16]**	**27 F**	**49**	**9 Y**
								**17 S**		**8 U**

ID fam, family code; MC, number of mutations carriers in the family; AOO, age of onset ranges in the family; DD, disease duration (at death or last examination); APOE, Apolipoprotein E genotype; F, familial; S, sporadic; Y, yes, U, unknown.

* Indicates a previously reported de novo mutation in a sporadic case [20, 21, 40].

**Table 2:** Previously unreported French families with AD-EOAD carrying a *PSEN2* mutation. Novel mutations appear in bold.

Protein change	Nucleotide change	Exon	Pathogenicity	APOE	ID fam	AOO (years)	DD (years)	Family history	MC (*n*)
p.Thr122Pro	c.364A>C	6	probable	E3 E4	EXT 441	[45–47]	[2–7]	F	1
**p.Arg284Gly**	**c.850A>G**	**9**	**possible**	**E3 E4**	**GRE 004**	**57**	**6**	**F**	**1**
**Total and ranges:**					**2**	**[45–57]**	**[2–7]**	**2 F**	**2**

ID fam, family code; MC, number of mutations carriers in the family; AOO, age of onset ranges in the family; DD, disease duration (at death or last examination); APOE, Apolipoprotein E genotype; F, familial; S, sporadic.

**Table 3:** Previously unreported French families with AD-EOAD carrying an *APP* mutation.

Protein change	Nucleotide change	Exon	Pathogenicity	APOE	ID fam	AOO (years)	DD (years)	Family history	MC (*n*)
p.Ala713Thr	c.2137G>A	17	definite	E3 E3	EXT 1064	50	3	F	1
				E2 E3	EXT 551	[62–64]	[2–3]	F	2
				E3 E3	ROU 1580	56	4	F	1
				E3 E3	EXT 1059	[61–66]	[4–9]	F	1
				E3 E4	ROU 1562	[50–85]	[5–9]	F	2
p.Val717Ile "London"	c.2149G>A	17	definite	E3 E3	ALZ 620	[50–52]	[2–8]	F	1
				E3 E3	ALZ 568	[50–53]	[4–15]	F	1
				E3 E4	EXT 1055	[45–50]	[3–4]	F	1
				E3 E4	EXT 1044	[48–55]	[2–6]	F	1
				E3 E4	EXT 1017	[40–50]	[2–4]	F	1
				E3 E3	EXT 1015	[48–60]	[3–5]	F	1
				E3 E4	EXT 993	[40–50]	[4–13]	F	1
				E3 E3	EXT 599	[39–61]	[3–9]	F	1
				E3 E3	EXT 519	[56–65]	[4–7]	F	1
				E3 E3	EXT 397	[50–56]	[4–10]	F	2
				E3 E3	SAL 638	[45–54]	[3–4]	F	1
p.Lys724Asn "Belgian"	c.2172G>C	17	definite	E3 E3	EXT 624	[55–65]	[7–14]	F	1
p.Asp694Asn "Iowa"	c.2080G>A	17	definite	E3 E3	EXT 233	[51–56]	[1–11]	F	2
p.Glu693Lys "Italian"	c.2077G>A	17	definite	E3 E3	EXT414	[60–63]	5	F	2
p.Ala692Gly "Flemish"	c.2075C>G	17	definite	E3 E3	EXT 1025	[45–51]	[2–9]	F	1
**Total and ranges:**					**20**	**[39–85]**	**[1–15]**	**20 F**	**25**

ID fam, family code; MC, number of mutations carriers in the family; AOO, age of onset ranges in the family; DD, disease duration (at death or last examination); APOE, Apolipoprotein E genotype; F, familial; S, sporadic.

**Table 4:** Previously unreported French families with AD-EOAD and sporadic cases carrying an *APP* duplication.

Protein change	Duplication size (Mb)	APOE	ID fam	AOO (years)	DD (years)	MC (*n*)	Family history	De Novo
DUP APP	2.2	E3 E3	EXT 1093	[53–65]	[6–9]	1	F	
DUP APP	1.4	E3 E4	EXT 857	[56–62]	[2–6]	1	F	
DUP APP	5.9	E3 E3	EXT 814	[50–54]	[8–10]	1	F	
DUP APP	1.4	E3 E3	EXT 1252	[54–58]	2	1	F	
DUP APP*	7.6	E3 E3	EXT 773	44	12	1	S	Y
**Total and ranges:**	**[1.4–7.6]**		**5**	**[44–65]**	**[2–12]**	**5**	**4F 1S**	

ID fam, family code; MC, number of mutations carriers in the family; AOO, age of onset ranges in the family; DD, disease duration (at death or last examination); APOE, Apolipoprotein E genotype; F, familial; S, sporadic.

* Indicates a previously reported de novo mutation in a sporadic case [20].

#### PSEN1

Five of the 12 novel *PSEN1* mutations were identified in AD-EOAD families: a sister and the mother of the patient carrying the c.251T>C, p.(Met84Thr) mutation were also affected with AD (age at death: 61 and 64 y, respectively); the father of the patient carrying the c.263C>A, p.(Pro88His) mutation died at age 47 with an AD diagnosis; the father of the patient carrying the c.629T>G, p.(Met210Arg) mutation died from AD at age 50, with an AOO of 47 y; the mother and the maternal grandmother of the patient carrying the c.1148T>G, p.(Leu383Trp) mutation died from AD at 54 and 50 y, respectively (AOO was 47 y for both). We also detected in an AD-EOAD family a novel genomic in-frame deletion encompassing *PSEN1* exons 9 and 10: c.(868+1_869–1)_(1129+1_1130–1)del, p.Ser290_Arg1129delinsTrp, thereafter named Δ9–10, which resulted in a missense change from serine to tryptophan at the aberrant exon 8–11 junction (Table 1). The remaining 7 novel *PSEN1* mutations were found in patients with sporadic EOAD. Among these mutations, a censoring effect was observed in families of patients carrying the c.772T>C, p.(Leu241Arg), the c.539T>A, p.(Ile180Asn), and the c.710T>G, p.(Phe237Cys) substitutions, while the c.331G>T, p.(Gly111Trp), the c.350C>A, p.(Pro117Gln), and the c.614_616del, p.(Phe205_Gly206delinsCys) mutations occurred de novo. The seventh patient carried the c.1078G>A p.(Ala360Thr) variant. No censoring effect was noted in his family, but parental DNA was not available to verify the de novo occurrence of the mutation (Table 1). Among carriers of the *PSEN1* mutation, the clinical presentation was mainly isolated progressive cognitive decline, but six patients carrying either the p.(Pro264Leu), p.(Leu173Trp), p.(Gln222His), or the Δ9–10 *PSEN1* mutation displayed an associated phenotype of spastic paraparesis. Another patient carrying the *PSEN1* p.(Gly378Glu) substitution also exhibited an atypical presentation: cerebellar ataxia and extra pyramidal syndrome.

#### PSEN2

Only one novel *PSEN2* mutation, c.850A>G, p.(Arg284Gly), and a previously known mutation, p.(Thr122Pro), were identified during this screen (Table 2). No atypical phenotype was noticed.

#### APP

In the *APP* gene, no novel mutation was found. We identified a previously reported mutation in 25 patients from 20 AD-EOAD families (Table 3). The most frequent one was the c.2149G>A, p.(Val717Ile) substitution, which was present in 12 subjects from 11 families. Clinical features were typical of AD with amnestic presentation. The c.2137G>A, p.(Ala713Thr) mutation was found in 7 patients from 5 unrelated families. They exhibited a progressive cognitive decline starting from age 50 to 66 y. Notably, the mother of a patient who carried the mutation together with an *APOE 4–4* genotype had no cognitive impairment until the age of 85, when she presented recurrent lobar hematoma. In addition, 5 subjects from 3 families carried mutations located within the coding sequence of the Aβ peptide: one carried the “Flemish” *APP* mutation c.2075C>G, p.(Ala692Gly), two carried the “Italian” mutation c.2077G>A, p.(Glu693Lys), and another two carried the “Iowa” mutation c.2080G>A, p.(Asp694Asn). A complete description of the phenotype of these 5 patients is provided in Sellal et al. [19].

#### APP duplications

Four subjects in four distinct AD-EOAD families and a sporadic case carried an *APP* duplication (Table 4). All patients exhibited progressive cognitive impairment. Only one presented signs of CAA and suffered from intracerebral hematoma at the age of 60.

#### CSF biomarkers

CSF biomarkers were available for 53 out of 81 mutation carriers (65%) (Table 5). There was no significant difference in Aβ_42_, Tau, and P-Tau mean values between patients bearing *PSEN1* and *APP* mutations or duplications (two groups, *p*-values = 0.78, 0.19, and 0.16, respectively, Mann–Whitney U test). Among the 53 patients, 46 (87%) were classified PLM 3, 5 (9%) were classified PLM 2, and 2 (4%) were classified PLM 1; no patient was classified PLM 0. Among the 5 patients classified PLM 2, 2 had low Aβ_42_ and elevated Tau levels, and 3 had elevated Tau and P-Tau with normal Aβ_42_ CSF level. Two of the latter 3 patients carried a *PSEN1* mutation: 1 carried the p.(Leu383Trp) with AOO at 57 y and 4 y of evolution, and the other carried the p.(Ala231Thr) with AOO at 50 y and 3 y of evolution. The third one carried an *APP* p.(Val717Ile) mutation with an AOO at 56 y and 4 y of evolution. The two patients classified PLM 1 had low Aβ_42_ value, without Tau or P-Tau elevation. One carried a p.(Ala360Thr) *PSEN1* mutation with AOO at 45 y and 3 y of evolution; the second carried a p.(Ala692Gly) *APP* mutation with AOO at 45 y and 2 y of evolution.

**Table 5:** CSF biomarkers levels in mutation carriers (pg/mL).

Gene	Mutation	ID	Aβ_42_	Tau	p-Tau	PLM
*PSEN1*	p.Ala79Val	EXT 85	**494**	**>1,200**	**206**	**3**
*PSEN1*	p.Thyr115Cys	EXT 755	**622**	**207**	**68**	**3**
*PSEN1*	p.Pro117Gln	EXT 851	**587**	**>1,200**	**173**	**3**
*PSEN1*	p.Ile143Thr	EXT 670	**393**	**>1,200**	**84**	**3**
*PSEN1*	p.Met146Ile	EXT 622	**543**	**857**	**105**	**3**
*PSEN1*	p.His163Arg	EXT 1242	**615**	**>1,200**	**129**	**3**
*PSEN1*	p.His163Arg	EXT 766	**434**	**849**	**66**	**3**
*PSEN1*	p.Phe205_Gly206 delinsCys	EXT 177	**376**	**397**	**91**	**3**
*PSEN1*	p.Met210Arg	EXT 832	**235**	**672**	**104**	**3**
*PSEN1*	p.Gln222His	EXT 807	**502**	**1,000**	**132**	**3**
*PSEN1*	p.Ala231Thr	EXT 680	772	**1,028**	**113**	**2**
*PSEN1*	p.Met233Thr	EXT 1201	**440**	**692**	**107**	**3**
*PSEN1*	p.Phe237Leu	EXT 1127	**430**	**>1,200**	**142**	**3**
*PSEN1*	p.Leu241Arg	EXT 504	**464**	**595**	**94**	**3**
*PSEN1*	p.Ala246Pro	EXT 1194	**470**	**523**	**80**	**3**
*PSEN1*	p.Cys263Phe	EXT 1193	**561**	**993**	**121**	**3**
*PSEN1*	p.Cys263Phe	EXT 768	**454**	**368**	**66**	**3**
*PSEN1*	p.Pro264Leu	EXT 966	**543**	**731**	**92**	**3**
*PSEN1*	p.Pro264Leu	EXT 1010	**445**	**696**	**92**	**3**
*PSEN1*	p.Arg269His	EXT 1228	**231**	**558**	**111**	**3**
*PSEN1*	p.Glu273Gly	EXT 886	**541**	**>1,200**	**165**	**3**
*PSEN1*	P.Glu273Gly	EXT 1195	**643**	**767**	**104**	**3**
*PSEN1*	p.Ala360Thr	SAL 629	**487**	217	35	1
*PSEN1*	p.Gly378Glu	EXT 390	**515**	**545**	**79**	**3**
*PSEN1*	p.Gly378Val	EXT 596 ind. 001	**288**	**522**	**86**	**3**
*PSEN1*	p.Gly378Val	EXT 596 ind. 002	**464**	**517**	**79**	**3**
*PSEN1*	p.Leu383Trp	EXT 1071	745	**1,140**	**130**	**2**
*PSEN1*	p.Val391Phe	EXT 902 ind. 001	**279**	**782**	**129**	**3**
*PSEN1*	p.Val391Phe	EXT 902 ind. 002	**545**	**495**	41	2
*PSEN1*	p.Ser290_Ser319delinsCys (Δ 9)	EXT 235	**481**	**777**	56	2
*PSEN1*	Δ exon 9–10	EXT 313	**153**	**414**	**64**	**3**
*APP*	p.Ala713Thr	EXT 1064	**344**	**>1,200**	**191**	**3**
*APP*	p.Ala713Thr	ROU 1580	**605**	**>1,200**	**229**	**3**
*APP*	p.Ala713Thr	EXT 551	**150**	**>1,200**	**150**	**3**
*APP*	p.Ala713Thr	EXT 1059	**246**	**>1,200**	**212**	**3**
*APP*	p.Ala713Thr	ROU 1562	**287**	**1,198**	**156**	**3**
*APP*	p.Val717Ile	ALZ 568	**252**	**809**	**118**	**3**
*APP*	p.Val717Ile	EXT 1055	**545**	**533**	**69**	**3**
*APP*	p.Val717Ile	EXT 1044	**603**	**841**	**102**	**3**
*APP*	p.Val717Ile	EXT 1017	**595**	**974**	**101**	**3**
*APP*	p.Val717Ile	EXT 1015	**663**	**>1,200**	**357**	**3**
*APP*	p.Val717Ile	EXT 993	**255**	**573**	**91**	**3**
*APP*	p.Val717Ile	EXT 519	**595**	**1,008**	**94**	**3**
*APP*	p.Val717Ile	EXT 397	801	**841**	**112**	**2**
*APP*	p.Val717Ile	SAL 638	**536**	**732**	**132**	**3**
*APP*	p.Lys724Asn	EXT 624	**427**	**720**	**74**	**3**
*APP*	p.Asp694Asn	EXT 233	**316**	**>1,200**	**203**	**3**
*APP*	p.Glu693Lys	EXT 414	**334**	**422**	**77**	**3**
*APP*	p.Ala692Gly	EXT 1025	**594**	226	45	1
*APP*	Duplication	EXT 857	**485**	**485**	**67**	**3**
*APP*	Duplication	EXT 814	**484**	**721**	**70**	**3**
*APP*	Duplication	EXT 1093	**311**	**808**	**93**	**3**
*APP*	Duplication	EXT 1252	**519**	**1,215**	**202**	**3**

ID, family code; ind, individual code. Abnormal values appear in bold.

>1,200: CSF Tau values higher than 1,200 but not diluted for a second dosage by the local center.

#### Neuropathology

Neuropathological examination was available for three subjects. For patient EXT 773, who carried an *APP* duplication, the diagnosis was definite AD with Braak stage VI, Thal stage V. There was amyloid deposition in vessel walls in the insula and basal ganglia. Signs of severe CAA were found in middle frontal gyrus, superior temporal gyrus, inferior parietal cortex, and primary motor area. Lewy bodies were found in the amygdala, locus niger, nucleus basalis of Meynert, and entorhinal cortex.

For patient EXT 149, who carried the c.518T>G, p.Leu173Trp de novo *PSEN1* mutation, rare senile plaques associated with numerous cotton wool deposits and neurofibrillary tangles were present in hippocampal regions and cortical areas. Lewy bodies were found in the amygdala and limbic cortex as well as the frontal, temporal, and parietal cortices and cingulum. CAA was noted in hippocampal regions, the temporal lobe, and the cerebellum.

For patient EXT 1117, who carried the c.251T>C, p.Met84Thr *PSEN1* mutation, neuropathological examination showed global atrophy, particularly in temporal lobes. Samples from the cerebellum and the frontal, temporal, and parietal cortices showed numerous senile plaques and neurofibrillary tangles associated with severe CAA. No Lewy bodies were observed.

### Mutational spectrum in the whole French EOAD series

Adding this sample to our previous reports [1,8,9,14,15,19,21–24], a total of 170 AD-EOAD families and 18 sporadic cases carrying mutations in genes known to cause EOAD have now been identified by our national reference center. Ninety distinct mutations (78 *PSEN1*, 4 *PSEN2*, and 8 *APP*, including *APP* duplication) were represented by respectively 127, 9, 34, and 18 occurrences in this whole sample (S1 Table). For each distinct mutation, the frequency reported in the Exome Aggregation Consortium (ExAC) database [25], which colligates human exome data from ~60,000 individuals, is null or very low (S1 Table).

The mean AOO for *PSEN1* mutation carriers was 44.4 y (range 24–80), 53.9 y (range 45–69) for *PSEN2* mutation carriers, 50.9 y (range 39–85) for *APP* mutation carriers, and 51.1 y (range 41–69) for patients carrying *APP* duplications. Variation of AOO by mutated gene was similar to the one reported by Ryman et al. (2014) [26].

### Sporadic cases and de novo mutations

Among the 129 patients with a sporadic presentation and an AOO before 51 y for whom a mutation screening was performed, we identified 18 mutations, including 17 *PSEN1* mutations and 1 *APP* duplication (Tables 1 and 4). For 10 patients, DNA of the unaffected parents was available, and analysis of parental DNA showed that the 10 mutations had occurred de novo: 7 patients carried a de novo *PSEN1* missense mutation, 1 carried a de novo splicing *PSEN1* mutation, 1 carried a de novo *PSEN1* indel, and another 1 carried an *APP* de novo duplication. Interestingly, 5 out of 7 missense de novo *PSEN1* mutations occurred at a position already known to be hit by pathogenic mutations. Parental DNA was not available for the remaining *PSEN1* mutation carriers, but we noted a strong censoring effect due to a young age at death in two families, and the parents were unknown in three other families. For the remaining 3 patients, the absence of both a censoring effect and AD history in the parents is suggestive of a de novo occurrence, but this could not be proved by parental DNA analysis.

## Discussion

We have studied two samples of EOAD patients and identified 10 novel missense mutations, 1 novel indel, and 1 novel genomic deletion in *PSEN1* and 1 novel missense mutation in *PSEN2*. According to the Guerreiro’s algorithm [12], pathogenicity was considered as definite for 1 mutation, probable for 9, and possible for 3. Considering the whole French EOAD series, 90 distinct mutations (including the *APP* duplication) are now reported, and pathogenicity is considered definite for 69 mutations (77%), probable for 16 (18%), and possible for 3 (5%).

The pathological effect of three known mutations deserves discussion because of incomplete penetrance, nonpathogenicity, or wide range of AOO.

The *PSEN1* c.236C>T, p.(Ala79Val) substitution is currently considered pathogenic and leads to an increase in Aβ_42_ level and Aβ_42_/Aβ_40_ ratios in cell cultures [27]. However, this variant seems to be associated with a later onset compared to the other *PSEN1* variants. It was found in several families with late-onset AD (LOAD) [26,28,29]. Four mutation carriers from one family had a definite, neuropathological diagnosis of AD and an AOO after 75 y [28]. Of note, this variant has been reported once in the ExAC database [25] (among ~60,000 controls). Considering that it was also found in subjects with EOAD [30,31], these data suggest that this mutation is associated with a large range of AOO (53–78 y), which could lead to underestimation of its frequency and is of importance for genetic counseling.

Second, the *PSEN2* c.211T>C, p.(Arg71Trp) variant was initially found in patients with LOAD [12,32,33]. We previously reported this variant in two EOAD families [14], but we removed it from our complete list because it is now considered as nonpathogenic. It did not segregate with AD in several families [32], including 8/14 affected individuals not carrying this variant in one large family [29]. It was found with an allele frequency of 0.034% (1.95% in the Finnish population) in the ExAC database. When coexpressed in HEK293 cells with *APP*, the variant did not alter the Aβ_42_/Aβ_40_ ratio in vitro [34]. As previously discussed, these elements lead us to consider this mutation as nonpathogenic [15].

Third, since our first report in a patient with sporadic probable AD [8], the *APP* c.2137 G>A p.(Ala713Thr) mutation has now been found in 24 patients from 11 families [5,35–39], including the 6 patients from 5 families included here. Although cerebrovascular lesions were described in brain imaging of some of these patients [36–38], the clinical presentation was a progressive cognitive decline in all but one of the reported cases. Interestingly, AOO ranged from 49 to 85 y, and several asymptomatic carriers were also reported, including one 88-y-old woman [8]. In one family, the mutation was found homozygous in 3 patients [38], and the disease onset was not different from the heterozygous carriers. In the present report, the mother of the proband ROU-1562 had no cognitive impairment until the age of 85, when a diagnosis of probable CAA was made. Of note, this variant has been reported with an allele frequency of 0.0058% in the ExAC database. Taken together, this suggests that the p.Ala713Thr substitution is a pathological variant with reduced penetrance, which is unusual compared to other *APP* mutations and is of main consequence for genetic counseling.

A notable finding of this study, as compared with the state of the literature, is the number (*n* = 10) of de novo *PSEN1* or *APP* mutations detected in this set of 129 sporadic cases with onset below age 51. Furthermore, this could be underestimated, as parental DNA was not available for all cases. To our knowledge, only four de novo mutations had previously been reported in *APP* or *PSEN1*, including three by our group [20,22,23,40]. To our knowledge, there is no evidence to suggest that the *PSEN1* gene is a hot spot of de novo mutations. Following the estimations by Samocha and coauthors [41] provided on the ExAC database [25], the probability to observe a *PSEN1* de novo missense mutation in an individual is 1.29 x 10^−5^. This probability is that of an average gene since 56% of genes are more mutable and 44% less mutable than *PSEN1*. Thus, the discrepancy between the low number of previously reported de novo *PSEN1* mutations in sporadic EOAD patients and the present report is likely to reflect a lack of inclusion of these patients in previous mutational screenings, which focused on familial cases. This underscores the need to systematically include patients with sporadic presentation and very early AOO in genetic screening. Consequences for genetic counseling are important, as the offspring of a mutation carrier has a same 50% risk to be a mutation carrier regardless of the familial or sporadic presentation of the affected parent; the offspring can then (i) be accurately informed, (ii) ask for a presymptomatic testing, and (iii) be a possible candidate for preventive clinical trials [13].

Concerning CSF biomarkers, 48/53 (91%) of patients with available CSF exhibit signs of both Aβ and Tau pathology, and 87% of the mutation carriers were classified PLM 3. This is higher than the 76% reported in our previous series [14]. This difference can be explained by the change in the Aβ_42_ cutoff (<700 versus <500 pg/ml in our previous series) according to the 2013 recommendations of the PLM network, whose aim is to homogenize preanalytical treatment for CSF biomarkers across French centers [42]. Overall, no AD mutation carrier presented with normal CSF biomarkers, suggesting that when all three CSF biomarkers are in normal ranges, genes involved in other neurodegenerative diseases should be screened in the first instance.

Our primary goal was to provide to clinicians a list of variants that can accurately be used in genetic counseling. Considering our whole series, this goal is achieved for 60/78 (77%) of *PSEN1*, 1/4 of *PSEN2*, and 8/8 of *APP* mutations reported in the French population. However, despite a large effort, too many mutations in AD-EOAD genes remain insufficiently characterized, and some are incompletely penetrant. The recent analysis of ~60,000 human exomes by the ExAC consortium has revealed an implausibly high per-individual burden of variants reported as causing disease in databases listing Mendelian disease alleles. These findings cast doubt on the validity of these databases and lead to a reclassification of numerous variants as benign [25]. In this context, it is reassuring to see that all variants reported here have a null or very low frequency in ExAC, which is a strong argument for pathogenicity.

A limitation of this study is the absence of functional assessment of the possibly and probably pathogenic variants, which should help their classification. Moreover, only three genes were analyzed. It is possible that de novo mutations in other genes are also involved in the genetic determinism of sporadic forms. To address this latter issue, the next step is now to perform exome sequencing on negatively screened families and sporadic cases. Indeed, this approach already enabled us to show that (i) rare variations in the *SORL1* gene might be responsible of a subset of AD-EOAD families [43] or at least constitute a penetrant risk factor for familial EOAD [44] and (ii) a set of genes defining an Aβ-centered genetic network are enriched in de novo mutations in sporadic cases [20].

Our findings suggest that a nonnegligible fraction of *PSEN1* mutations occur de novo. The practical implication for clinicians is to highlight the need to systematically include patients with sporadic presentation and very early AOO in genetic screening for the *APP*, *PSEN1*, and *PSEN2* genes. In addition, the need to pursue the effort to classify variants should be emphasized since, based on our results, definite pathogenicity is currently established for only 77% of identified mutations in these genes.

## Supporting information

S1 Table Different *PSEN1* (*n* = 78), *PSEN2* (*n* = 4), and *APP* (n = 8) mutations identified in the French EOAD whole series, totalizing 188 occurrences. Y = yes.
(XLSX)

S1 Analysis plan Analysis plan. (DOCX)

## Abbreviations

Aβ: amyloid β
AD: Alzheimer disease
AD-EOAD: autosomal dominant early-onset Alzheimer disease
AOO: age of onset
APOE: *Apolipoprotein E*
*APP*: *amyloid protein precursor*
CAA: cerebral amyloid angiopathy
CSF: cerebrospinal fluid
EOAD: early-onset Alzheimer disease
ExAC: Exome Aggregation Consortium
LOAD: late-onset Alzheimer disease
PLM: Paris, Lyon, Marseille
*PSEN1*: *presenilin-1*
*PSEN2*: *presenilin-2*
P-Tau: phospho-tau protein
Tau: total tau protein
