# Supplementary material for: APP, PSEN1, and PSEN2 mutations in early-onset Alzheimer disease: A genetic screening study of familial and sporadic cases
Source: PLoS Med. 2017 Mar 28;14(3):e1002270. doi: 10.1371/journal.pmed.1002270 (PMC5370101; doi:10.1371/journal.pmed.1002270)
Supplement: S1 Analysis plan — (DOCX) [file pmed.1002270.s002.docx]

**Analysis plan**

**Mutational screening of *APP*, *PSEN1* and *PSEN2* genes in EOAD French patients.**

The original analysis plan was established in 2012 (see reference [1], Wallon et al., JAD, 2012). An update was planned every 5 years.

Systematic screening of a group of sporadic cases was further decided upon based on previous reports showing *de novo* *PSEN1* mutations and *APP* duplications (see references [2] and [3]).

**Aims of the study:**

- Providing an update of the French cohort of autosomal dominant early-onset families
- Identification of novel mutations
- Assessment of the pathogenicity of the mutations using Guerreiro’s scale.
- Inclusion of a group of sporadic cases to specifically identify *de novo* mutations in this group.

Implications for genetic counselling will be discussed.

**Study Plan:**

- Mutational screening of *APP*, *PSEN1* and *PSEN2* genes will be performed on EOAD subjects referred to the National Reference Centre for Early-Onset Alzheimer Patient (CNR-MAJ) from 28 University Hospitals across France.
- Search for mutations in *APP*, *PSEN1* and *PSEN2* genes on DNA extracted from blood cells will be performed (i) in families suggestive of autosomal dominant-EOAD presentation, i.e. if at least two first-degree relatives suffer from EOAD (age of onset < 65 years) in two generations or (ii) in sporadic presentations if a patient without family history of AD had an age of onset before 51 years.
- For each patient, AD diagnosis will be established using the National Institute of Aging-Alzheimer’s Association (NIA-AA) criteria. No other exclusion criteria will be applied.
- Familial cases will be those referred in the 2012-2016 interval (update of the previous sample).
- Sporadic cases will be those included from 1999 onwards.
- Mutations identified will be reported along with phenotypes. Mutations will be classified as novel or previously reported. The proportion of mutations classified as definitely pathogenic according to the Gueirrero’s algorithm will be reported
- When available, CSF biomarkers or neuropathological data will be collected and reported.

**References**

1. Wallon D, Rousseau S, Rovelet-Lecrux A, Quillard-Muraine M, Guyant-Marechal L, Martinaud O, et al. The French series of autosomal dominant early onset Alzheimer's disease cases: mutation spectrum and cerebrospinal fluid biomarkers. Journal of Alzheimer's disease : JAD. 2012;30(4):847-56.

2. Rovelet-Lecrux A, Charbonnier C, Wallon D, Nicolas G, Seaman MN, Pottier C, et al. De novo deleterious genetic variations target a biological network centered on Abeta peptide in early-onset Alzheimer disease. Molecular psychiatry. 2015;20(9):1046-56.

3. Dumanchin C, Brice A, Campion D, Hannequin D, Martin C, Moreau V, et al. De novo presenilin 1 mutations are rare in clinically sporadic, early onset Alzheimer's disease cases. French Alzheimer's Disease Study Group. Journal of medical genetics. 1998;35(8):672-3.
